# *Demodex* Mite Infestation in Patients Suffering from Atopic Dermatitis and Psoriasis

**DOI:** 10.3390/pathogens14090956

**Published:** 2025-09-22

**Authors:** Agnieszka Borzęcka-Sapko, Magdalena Raszewska-Famielec, Aleksandra Sędzikowska, Alicja Buczek, Adam Wilmosz Borzęcki, Katarzyna Bartosik

**Affiliations:** 1 Department of Biology and Parasitology, Chair of Pharmacology and Biology, Faculty of Health Sciences, Medical University of Lublin, Radziwiłłowska 11 St., 20-080 Lublin, Poland; alicja.buczek@umlub.edu.pl; 2 Med-Laser Non-Public Health Care Centre, 20-406 Lublin, Poland; laserlub@poczta.onet.pl; 3 Faculty of Physical Education and Health, University of Physical Education, 21-500 Biała Podlaska, Poland; raszewska.famielec@gmail.com; 4 Chair and Department of General Biology and Parasitology, Faculty of Medicine, Medical University of Warsaw, Chalubinskiego 5, 02-004 Warsaw, Poland; aleksandra.sedzikowska@wum.edu.pl

**Keywords:** *Demodex*, atopic dermatitis, psoriasis, emollients, PUVA, UVB

## Abstract

The role of *Demodex* mites in the pathogenesis of skin diseases still needs updating. The aim of the study was to determine the relationship between *Demodex* sp. infestation and ocular and skin lesions in patients with atopic dermatitis (AD) and psoriasis (PsO). The control group comprised subjects that had not been diagnosed with any inflammatory skin disease. Direct microscopic examination (DME) was applied to analyze eyelashes and skin scraping samples. *Demodex* mites were detected more often in the eyelash samples than in the skin in both the AD (16.7% vs. 6.7%) and PsO (18.5% vs. 7.4%) patients as well as in the control group (25.8% vs. 3%). The mean intensity of *Demodex* infestation was 4.25 in the AD patients, 3.4 in the PsO patients, and 2.8 in the control group. Emollients were used in the treatment by 76.7% of the AD patients and 64.8% of patients with PsO; however, this type of skin care did not significantly increase the risk of skin colonization by *Demodex*. AD and PsO do not seem to facilitate excessive *Demodex* sp. proliferation. Nevertheless, diagnosis of the presence of these mites should be considered in patients with facial skin and ocular lesions that do not respond to the treatment.

## 1. Introduction

Atopic dermatitis (AD), also known as atopic eczema, is a chronic and recurrent inflammatory disease. It is the most prevalent dermatosis with a significant impact on patient quality of life [1]. The disease manifests clinically as erythematous lesions with eczema-like morphology appearing in age-specific locations. Severe pruritus and dry skin are the main symptoms accompanying AD. The condition progresses with periods of remission and exacerbation [2,3]. In recent years, a significant increase in the incidence of the disease has been observed. The greatest increase in AD incidence is found in populations living in large cities in industrialized countries. In contrast, the opposite trend is observed in rural areas. The disease is estimated to affect approximately 0.9–22.5% of children and 2.1–8.1% of adults [4,5]. The first skin lesions in the course of the disease usually appear before the age of 1 year in approximately 50% of patients [6]. The etiopathogenesis of AD is extremely complex. The development of lesions is believed to involve genetic factors, immunological disorders, functional and biochemical skin defects, and environmental factors (e.g., food and airborne allergens) [7]. The genetic determinants of AD are corroborated by the increased tendency of the disease to affect families with a history of atopic conditions. Association with AD in various populations has been demonstrated for over 60 different genes [8]. The filaggrin gene (*FLG*) is currently recognized as one of the main genes contributing to AD activity. The metabolic products of filaggrin determine the maintenance of the acidic pH of the epidermis and serve as substrates for the synthesis of natural moisturizing factor (NMF). The absence of proper filaggrin gene expression causes excessive water loss and skin dryness, which can be observed in AD patients [9]. Another gene playing an important role in the pathogenesis of AD is *SPINK*, which encodes the LEKT1 protein, i.e., a universal inhibitor of epidermal serine proteases [10]. LEKT1 inhibitor deficiency increases the activity of endogenous and exogenous serine proteases in the epidermis, such as those secreted by *Staphylococcus aureus*. This results in the destruction of tight junctions between epidermal cells and the opening of the epidermal barrier to allergens and pathogens, which contribute to the development of AD-related inflammatory lesions [11].

The combination of physical and chemical barrier defects facilitates infectious complications of AD caused by, e.g., *S. aureus*, *Malassezia* spp., *Pityrosporum orbiculare*, herpes simplex virus (HSV-1), and molluscum contagiosum virus (MCV) [12,13]. One of the most common clinical symptoms of AD is persistent pruritus. A role in its pathogenesis is played by not only histamine but also many other factors, e.g., cytokines (IL-31, IL-33, thymic stromal lymphopoietin- TSLP), neuropeptides (SP, somatostatin), PAR-2 receptor activators (tryptase), opioids, and serotonin [14,15]. Reduced epidermal ceramide concentrations, impairing the barrier properties of the stratum corneum, have been demonstrated in AD patients. The reduction in the epidermal ceramide concentration promotes transdermal water loss and reduces epidermal elasticity, leading to increased susceptibility to micro-injuries and the penetration of pathogens per cutis [16,17]. Skin lesions in AD can be acute, subacute, or chronic. The primary lesion is an exudative papule on an erythematous base. Acute lesions are characterized by intense erythema, numerous papules, and vesicles with signs of exudation. Subacute lesions are characterized by excoriation and desquamation. Chronic lesions exhibit signs of lichenification [18].

Psoriasis (PsO), similar to AD, is a chronic and recurrent inflammatory skin disease. Its incidence varies depending on the geographical location or ethnicity. It occurs worldwide, affecting approximately 1–3% of the population [19]. The burden of PsO is highest among individuals aged 60 to 69 years, with comparable prevalence between men and women. This burden is disproportionately elevated in high-income countries and those with a high Socio-demographic Index (SDI) in North America and Europe. Objective assessment of PsO at the population level remains crucial [20]. The first symptoms of the disease may appear at any age, with two peaks of incidence: between 15 and 25 years of age and in over 40-year-old subjects [21]. Taking into account the age, the family history, the presence of histocompatibility antigens (HLA), and the course of the disease, two types of PsO have been distinguished. Hereditary PsO (Type I) manifests at a young age, often affects other family members, and is associated with the HLA-Cw6 antigen. Its course is associated with emergence of severe skin lesions and frequent exacerbations. Sporadic PsO (Type II) usually develops between 40 and 60 years of age, often without a known family history. The clinical picture is characterized by the presence of single erythematous-infiltrative plaques on the skin of elbows, knees, scalp, sometimes shins, and in the sacral region. This type of PsO is associated with antigens HLA-Cw2 and B27. Its course is stable, with rare exacerbations [22].

The etiopathogenesis of PsO is multifactorial, with immunological and epidermal cell proliferation disorders playing a central role [23]. Psoriasis is a polygenically inherited disease. At least 15 gene loci (psoriasis susceptibility locus; *PSORS15*) responsible for susceptibility to PsO have been identified [24]. Currently, *PSORS1* is the strongest known genetic determinant of the disease. HLA-Cw6 is the major susceptibility allele for PsO within *PSORS1* [25].

The majority of PsO cases correspond to chronic plaque-type PsO. The clinical manifestations are sharply demarcated erythematous plaques covered by silvery scales. The most common locations include the trunk, the extensor surfaces of the limbs, and the scalp [26]. The nail plates are also often affected by the disease process [27].

*Demodex* spp. (Acari: Demodecidae) are obligatory and permanent parasites of humans and many mammalian species. A characteristic feature of these mites is their high host specificity [28,29]. Two species have been identified as human parasites to date: *Demodex folliculorum* and *Demodex brevis*. On the human body, *Demodex* mites typically colonize sebaceous glands and hair follicles in the skin, especially in regions with increased sebum secretion—the forehead, chin, cheeks, nose, and external ear canals. They feed on the epithelial cells of sebaceous glands or hair follicles, sebum, lymph, and blood plasma [30].

Both *Demodex* species parasitizing humans are widespread in human populations. However, *D. folliculorum* is much more prevalent in human subjects, as demonstrated by examinations of, e.g., eyelash follicles. Cases of mixed infestations, i.e., the presence of both *Demodex* species in a single patient, are reported frequently as well [31,32,33]. Considering various determinants of the spread of *Demodex* infestations, a positive correlation between the presence of *Demodex* mites and the age of infested patients has been documented [34,35,36,37].

The skin type has also been shown as an important factor in *Demodex* infestations; subjects with oily or combination skin are more susceptible to the infestation than those with dry or normal skin. Subjects with oily skin are at risk of a higher prevalence of *Demodex* mites and greater numbers of infesting *D. folliculorum* specimens [32,33,35,38].

The minimum number of mites required to cause disease symptoms has not yet been precisely determined. Some diagnosticians propose that five *Demodex* sp. specimens per 1 cm^2^ of the skin is the threshold value, above which the intensity of *Demodex* infestation translates into clinical changes [32,39,40]. Cutaneous demodicosis is characterized by dry skin with milky-brown discolorations, erythematous papules, or pustules. Desquamation of the epidermis and inflammation of hair follicles, along with keratinization of their openings, are also observed [41]. Eyelid demodicosis is associated with redness of the eyelids and conjunctiva, itching, burning, watery eyes, and loss of eyebrows and eyelashes [32]. Clinical data link *Demodex* infestation with Meibomian gland dysfunction, resulting in instability of the tear film, blepharitis, and chalazion [42,43,44]. Cylindrical dandruff appears to be a pathognomonic symptom of ocular demodicosis [45].

Bacterial antigens present on the surface of *Demodex* mites can trigger an inflammatory response in the host; additionally, *Bacillus oleronius* bacteria present in the intestine of these parasites are implicated in the development of pathogenic inflammation and angiogenesis [46,47]. *Demodex* mites also participate in the transmission of *Streptococcus* spp. and *Staphylococcus* spp., which can cause inflammatory processes in surrounding tissues [48].

*Demodex* infestations are detected more frequently in patients with compromised systemic or local immunity. This relationship has been observed in, e.g., HIV-infected and AIDS patients [49,50], cancer patients [51,52], subjects with chronic renal failure undergoing dialysis [53], and patients undergoing PUVA/UVB phototherapy [54].

AD and PsO alter skin immunology towards hyperreactivity. The treatment of these diseases involves the use of anti-inflammatory drugs, including immunosuppressants with systemic and local effects. There are only few reports on *Demodex* infestations in AD and PsO patients, with inconclusive results. Therefore, the aim of this study was to examine the prevalence of *Demodex* sp. infestation in AD and PsO patients, which may contribute to elucidation of the impact of the parasites on the severity of lesions in these patients.

## 2. Material and Methods

### 2.1. Ethical Approval

The approval to conduct research on patients with AD and PsO was obtained from the Bioethics Committee at the Medical University of Lublin (approval number: KE-0254/345/2018). 

### 2.2. Study Group

The study group comprised patients who reported to the NZOZ Med-Laser Centre for treatment of lesions in the course of AD and PsO and were suspected with *Demodex* sp. infestation. The inclusion criteria were the aforementioned inflammatory diseases, regardless of the duration of the disease or the degree of symptom exacerbation. The exclusion criteria were pregnancy and breastfeeding. Information regarding medical treatment within 3 months preceding the study was collected based on patients’ medical histories. The control group consisted of dermatological patients without diagnosed chronic inflammatory diseases, i.e., AD and PsO, who were examined for the presence of *Demodex* sp. Only adults of both sexes with legal capacity were eligible for the study.

### 2.3. Parasitological Studies

Direct microscopic examination (DME) was conducted to assess the presence and density of *Demodex* mites in the collected samples [55]. The study consisted in collection of several eyelashes from patients’ left and right eyes and facial skin scrapings. Since the study included patients with inflammatory skin diseases (AD and PsO), diagnostic methods that give the lowest risk of skin irritation were selected. Two to three eyelashes were collected using sterile disposable tweezers (Polmil, Bydgoszcz, Poland). Facial samples were collected using sterile disposable carbon steel surgical blades (Swann Morton, Sheffield UK) after gently squeezing the contents of small comedones from an area of approximately 1 cm^2^ of skin. The eyelashes and skin scrapings were placed on a glass slide in Hoyer Medium prepared as proposed by Dubinina [56]. The preparations were assessed under an Olympus CX21 light microscope (Olumpus Tokyo, Japan) at 100–600× magnification. Detection of at least one adult or juvenile *Demodex* specimens or their eggs in the examined material was regarded as a positive result. Attention was also focused on the number and individual developmental forms of *Demodex* sp. in the slides. Intensity of infestation (also expressed as mite density) was quantified as the number of mites per preparation containing eyelashes or epidermal scrapings from a 1 cm^2^ area.

### 2.4. Statistical Analysis

Categorical (qualitative) variables were described using numbers (n) and frequencies (%). Measurable variables were described using the arithmetic mean (M), standard deviation (SD), median (Me), and minimum (min) and maximum (max) values.

Pearson’s chi-square test was used to analyze the relationships between the categorical variables. In the case of insufficient expected sample sizes in a 2 × 2 contingency table, the chi-square test with Yates’ correction was used. In turn, Fisher’s exact test was used when the sample size was less than 40, and the ML (maximum likelihood) chi-square test was used in contingency tables larger than 2 × 2.

The Z test for two independent proportions was used to compare the percentages in the two groups. The Mann–Whitney U test was used to test the significance of differences in the measurable variables in the two groups when the variables were not normally distributed or were discrete.

Additionally, logistic regression analysis was performed to assess the impact of independent variables, i.e., age, gender, and immunosuppressive treatment, on the likelihood of demodicosis occurrence in the studied patients.

A *p* value of <0.05 was considered statistically significant. The statistical calculations were performed using the STATISTICA 10 PL statistical package (TIBCO Software, Palo Alto, CA, USA).

## 3. Results

### 3.1. Characteristics of the Study Group

In total, 150 patients were enrolled in the study. The description of the study group in terms of patients’ sex and age and hospitalization due to AD and PsO (study group) and other diseases (control group) is presented in Table 1.

The skin lesions in the group of patients with inflammatory skin diseases, i.e., pruritus, redness, dryness, scaling, and exudation, varied in their location. Facial skin lesions were observed in 63.3% of the AD patients (19/30), in 24.1% of the patients (13/54) in the PsO group, and in 10.6% of the patients (7/66) in the control group. Pain or persistent itching caused by the skin lesions was reported by 90.0% of the AD patients (27/30) and 75.9% of the PsO patients (41/54).

The incidence of ocular symptoms in the groups differed slightly: 26.6% in the AD patients (8/30), 61.1% in the PsO patients (33/54), and 21.2% in the control group (14/66). None of the patients with the inflammatory skin diseases and ophthalmic symptoms used eye drops or other ophthalmic preparations to alleviate these symptoms. The co-occurrence of skin and ocular lesions was observed in 50.0% (15/30), 9.3% (5/54), and 3.0% (2/66) of the AD and PsO patients and in the control group, respectively. The AD patients most frequently reported exacerbations of the inflammatory diseases, which occurred once a week 50.0% (15/30) or once a month 20% (6/15). In turn, 74.1% of the PsO patients experienced exacerbations several times a year (40/54).

Oral immunosuppressive medications, i.e., systemic cyclosporine A treatment, were periodically administered to 7.4% of the studied PsO patients. Topical therapies, i.e., a calcineurin inhibitor (tacrolimus 0.1%) or corticosteroids (clobetasol propionate 0.05%), were administered to the analyzed patients most frequently. Localized immunosuppressive medication was applied as part of the therapy in 43.3% (13/30) of the AD patients and 31.5% (17/54) of the PsO patients. Psoralen combined with UV-A PUVA or UVB phototherapy was administered to 43.3% (13/30) of the AD patients and 63.0% (34/54) of the PsO group. This type of therapy was used in only 6.1% (4/66) of the control patients. Emollients were a crucial element of therapy administered to all the analyzed patients. They were continuously used by 76.7% (23/30) of the AD patients and 64.8% (35/54) of the PsO group. No improvement in the skin condition after the incorporation of emollients into the therapy was reported by 21.7% (5/30) of the AD group and 22.2% (12/54) of the PsO patients.

### 3.2. Demodex sp. Infestation

**Figure 1:**
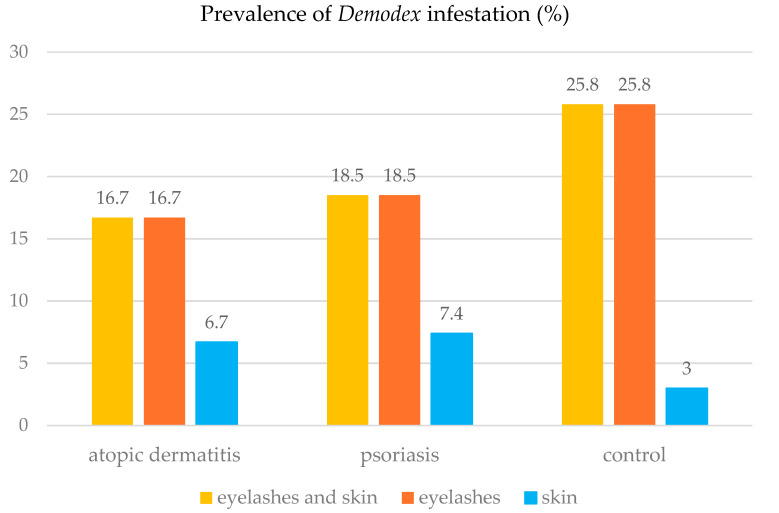
Prevalence of *Demodex* sp. infestation in the studied patients, depending on the type of the diagnostic material.

**Figure 2:**
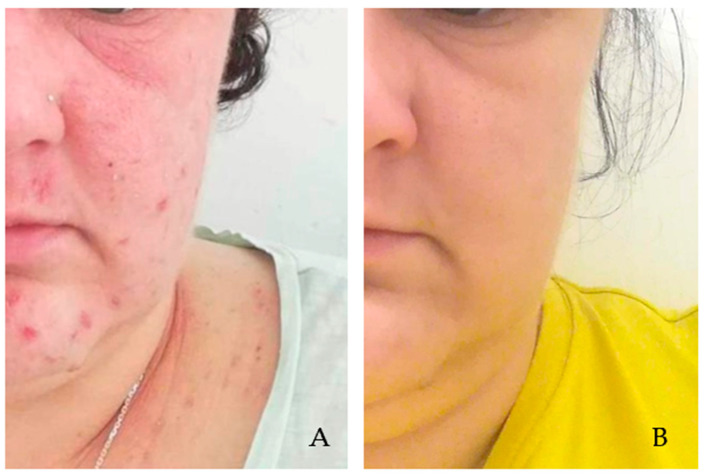
Demodicosis patient with AD exacerbation, with visible erythematous lesions not clearly demarcated from the surrounding area in the right and left periorbital regions and with isolated papular eruptions on facial skin (**A**). Picture after metronidazole therapy (500 mg/day for 10 days) and treatment with topical preparations (0.1% fludrocortisone acetate ophthalmic ointment, 0.1% methylprednisolone aceponate, 1% ivermectin cream, and 0.1% tacrolimus ointment—12 weeks). Significant improvement in the local condition is visible (**B**). Photograph: Agnieszka Borzęcka-Sapko.

**Figure 3:**
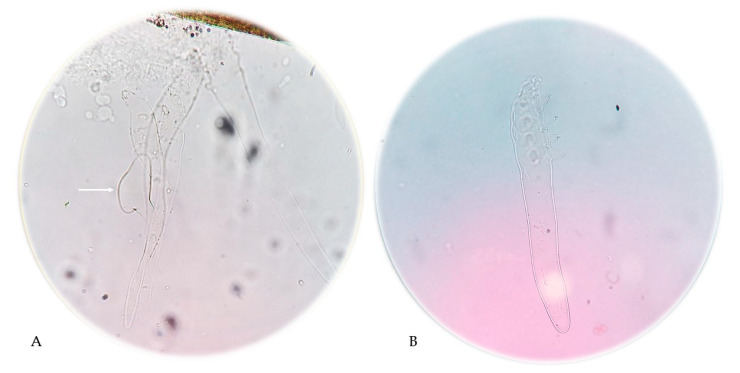
*Demodex folliculorum* mites present in the eyelash preparation. The arrow indicates a *Demodex* egg (**A**) and skin scrapings from the AD patient shown in Figure 1; (400× magnification). Photographs: Katarzyna Bartosik (**A**) and Agnieszka Borzęcka-Sapko (**B**).

In the group of patients with AD, *Demodex* mites were most frequently detected in the eyelash preparations. They were also detected in the skin of AD patients with concurrent infestation of eyelashes, as in the group of patients with general inflammatory skin diseases (Figure 1, Figure 2 and Figure 3).

In the group of the PsO patients, *Demodex* mites were most frequently present in the eyelash preparations (n = 10). In patients with *Demodex* mites detected in the skin (n = 4), these parasites were also found in the eyelash preparation (Figure 1). In the control group, *Demodex* mites were most frequently detected in the eyelash preparation (n = 17). In addition to the eyelash preparation, the mites were detected in the skin of some patients in this group (n = 2) (Figure 1).

The Z test for two independent proportions showed no significant difference in the prevalence of *Demodex* sp. between the control group and the patients with AD (Z = 0.72; *p* = 0.4713) or between the control group and the patients with PsO (Z = 0.73; *p* = 0.4684).

The numbers of *Demodex* mites detected in a single sample from an infested patient were in the following range:

– from 1 to 7 (mean 4.25) in the AD patients
– from 1 to 6 (mean 3.4) in the PsO patients
– from 1 to 8 (mean 2.8) in the control group

Although the mean number of *Demodex* mites in the samples from the AD and PsO patients was higher than in the control group, the Mann–Whitney U test did not demonstrate any significant differences (Z = 0.83; *p* = 0.4049, and Z = 0.77; *p* = 0.4398, respectively). The *Demodex* mites detected in all the infested patients were identified as *D. folliculorum*. The eyelash specimen collected from one PsO patient was indeterminate due to the location and fragmentation of the mite. Adult stages were detected most frequently, whereas *Demodex* eggs were the least common stage found in the samples.

#### 3.2.1. Sex

**Figure 4:**
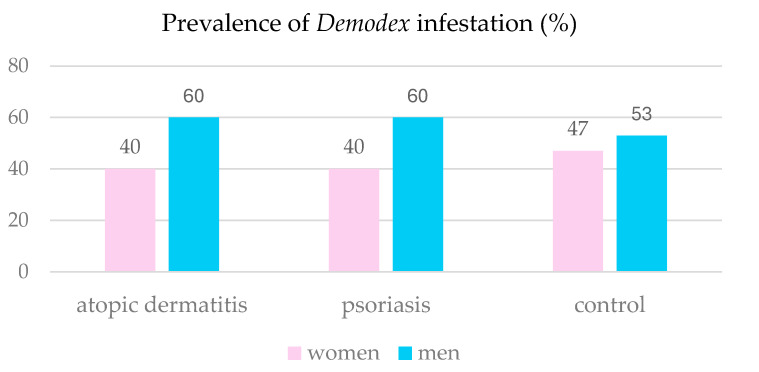
Prevalence of *Demodex* infestation in the patients studied in relation to the sex.

The association between the prevalence of *Demodex* sp. in the AD patients and their sex was examined using Fisher’s exact test. The analysis revealed no significant association between the sex and *Demodex* infestation in this group of patients (*p* = 0.6278). A similar analysis using the chi-square test with Yates’ correction was performed for the PsO patients. Again, no association between the *Demodex* sp. infestation and sex was found (*p* = 0.9688). Figure 4 shows the prevalence of *Demodex* sp. infestation in females and males from the study groups.

#### 3.2.2. Age

The mean and median for age in all the study groups were higher in those with the *Demodex* infestation. These values in the *Demodex*-infested AD patients were M = 48.6 years and Me = 43.0 years (SD = 21.9), respectively, while these values in the *Demodex*-non-infested group were M = 31.4 years and Me = 30.0 years (SD = 10.9), respectively. In the AD patient group, *Demodex* mites were detected in subjects aged from 22 to 80 years. In the PsO patients, the mean, median, and standard deviation for age were M = 50.1 years and Me = 50.0 years (SD = 11.8) in the *Demodex*-infested subjects and M = 45.8 years and Me = 45.0 years (SD = 13.2), respectively, in the non-infested group. *Demodex folliculorum* was detected in patients aged from 25 to 65 years in this group. Similar results were obtained in the control group, where these values were higher in subjects infested with *Demodex* sp., i.e., M = 50.2 years and Me = 51.0 years (SD = 18.2), than in the non-infested group, M = 42.9 years and Me = 45.0 years (SD = 18.6), respectively. The age of the infested subjects in this group ranged from 20 to 72 years. The discrepancies were analyzed using the Mann–Whitney U test, which revealed a significant difference in age between the *Demodex* sp.-infested and non-infested participants only in the inflammatory skin disease group, i.e., AD and PsO combined (*p* = 0.0311).

#### 3.2.3. Time of Onset of Inflammatory Skin Disease

The impact of the time of the onset of inflammatory skin disease on the risk of *Demodex* colonization was assessed using the NW chi-square test. The test did not show a significant correlation between the time of the appearance of the first disease symptoms and the risk of skin colonization by *Demodex* mites in the AD or PsO patients (Table 2 and Table 3).

#### 3.2.4. Exacerbation of Inflammatory Skin Diseases and *Demodex* Infestation

The frequency of periods with exacerbated disease symptoms was analyzed in relation to the presence of *Demodex* mites. Fisher’s exact test did not reveal a significant relationship between the frequency of disease exacerbation in the AD patients and the presence of *Demodex* sp. in this group (*p* > 0.05). Similarly, the chi-square test with Yates’ correction performed in the group of the PsO patients did not reveal a significant relationship between the frequency of disease exacerbations and *Demodex* infestations (*p* > 0.05). Pearson’s chi-square test employed in the analysis of patients with inflammatory skin diseases did not show a significant relationship between the frequency of disease exacerbation and the presence of *Demodex* sp. (*p* > 0.05) (Table 4 and Table 5).

The logistic regression model applied to analyze *Demodex* infestation in the context of sex, age, and immunosuppressive medication was significant at *p* = 0.0106, and its fit was as follows: Pseudo R^2^ = 0.070, R^2^ (Nagelkerke) = 0.109, and R^2^ (Cox-Snell) = 0.070. The age variable was found to be statistically significant (*p* = 0.0101), i.e., the older the patient, the greater the probability of *Demodex* infestation (Table 6).

#### 3.2.5. PUVA/UVB Therapy

The prevalence of *Demodex* mites in the AD patients in relation to the use of PUVA/UVB therapy was analyzed using Fisher’s exact test. The statistical analysis did not reveal a significant relationship between the use of PUVA/UVB therapy and the prevalence of *Demodex* sp. (*p* > 0.05) (Table 7).

Similarly, the chi-square test with Yates’ correction used in a similar analysis of the PsO group did not reveal a significant relationship between the application of phototherapy and *Demodex* infestation in these patients (*p* > 0.05) (Table 8).

#### 3.2.6. Emollients

The statistical analysis showed that emollient-based skin care was used significantly more frequently by the AD and PsO patients than in the control group (Z test for two independent proportions, *p* < 0.0001, and Z test for two independent proportions, *p* < 0.0001, respectively). The prevalence of *Demodex* mites in the AD patients who used emollients and those who did not use these agents was similar, i.e., 14.3% vs. 17.4%, respectively. Fisher’s exact test did not reveal a significant relationship between the use of emollients by the patients with AD and the prevalence of *Demodex* sp. (*p* = 1.0000). In contrast, the percentage of *Demodex* mite infestations in PsO patients who used emollients was almost twofold higher than that in the patients that did not use any emollients, i.e., 26.3% and 14.3%, respectively. Nevertheless, the chi-square test with Yates’ correction did not reveal a significant relationship between the use of emollients by the PsO patients and the prevalence of *Demodex* mites (χ^2^ = 0.52; *p* = 0.4715).

#### 3.2.7. Ophthalmic Symptoms

The relationship between the *Demodex* mite prevalence and ophthalmic lesions in the patient groups was analyzed using statistical tests. The results from the AD and PsO groups were analyzed with Fisher’s exact test and the chi-square test with Yates’ correction, respectively. Fisher’s exact test did not reveal a significant relationship between the ophthalmic symptoms in the AD patients and the prevalence of *Demodex* mites (*p* > 0.05) (Table 9). Similarly, the chi-square test with Yates’ correction and Pearson’s chi-square test did not reveal a significant correlation between the ophthalmic symptoms and the prevalence of *Demodex* sp. in the PsO patients (*p* > 0.05) (Table 10).

## 4. Discussion

It was observed during this study that the use of systemic immunosuppressive medications in AD and PsO patients is not common practice. In both groups, the majority of the patients used medicated skin creams or ointments: 43.3% in AD and 31.5% in PsO. This suggests that topical treatment is preferred over systemic therapy, which may be related to the fewer side effects and better tolerability of this form of therapy as well as the specificity of the patient group, including the severity of their disease. Noteworthy, none of the patients used eye drops or ointments, even despite the presence of bothersome ocular conditions, such as *blepharitis*. These lesions may be associated by PsO or AD patients with the course of the primary disease, whereas this study showed that the ocular lesions in 18.5% of the PsO patients and 16.7% of the AD patients may have been caused by the *Demodex* infestation itself or had a mixed etiology, which should be considered in ophthalmic treatment strategies for such patients. All the patients with ocular symptoms and *Demodex* infestation experienced alleviation of symptoms upon treatment targeted at *Demodex* mites. The most common treatment regimen was a combination therapy comprising 250 mg of oral metronidazole administered three times daily for 14 days and topical 1% ivermectin cream in the case of skin infestation. The patients were also advised to use moisturizing eye drops containing hyaluronic acid.

In the study group, 76.7% and 64.8% of the AD and PsO patients, respectively, used emollients as part of their therapy. These formulations help to seal and rebuild the hydrolipid barrier of the skin, reduce the severity of pruritus, improve overall skin hydration, and reduce the frequency and severity of exacerbations of these conditions [57,58]. In their study of the effect of the sebum composition in patients infested with *Demodex* sp. and in the control group, Demirdağ et al. detected significantly higher levels of cholesterol oleate in the sebum of patients infested by *Demodex* mites. The researchers suggested that cholesterol oleate, i.e., a natural emollient, may facilitate the proliferation of *Demodex* mites [59]. In contrast, the present study did not demonstrate a relationship between emollient-based skin care and the prevalence and severity of *Demodex* infestations. Therefore, the emollients used by the analyzed patients probably did not favor skin colonization by these parasites. However, during the emollient therapy, it is important to maintain good hygiene and avoid sharing these agents with other family members, in particular in the case of formulations that are not applied in a hygienic way (e.g., they are taken with fingers from a screw-top container). *Demodex* mites can survive in cosmetics, which may promote their indirect transmission [60].

In the analyzed group, 43.3% and 63.0% of the AD and PsO patients, respectively, were treated with PUVA/UVB phototherapy. There are only few scientific reports on the effect of phototherapy on *Demodex* sp. colonization. Aytekin [61] described a case of intense infestation by these mites in a PsO patient undergoing UVB 311 nm phototherapy. As reported by Kulac et al., *D. folliculorum* was detected four times as frequently in patients undergoing phototherapy than in the control group, i.e., 28.9% vs. 7.0%, respectively [54]. The researchers examined PsO and vitiligo patients who received PUVA and UVB 311 nm therapy. Demodicosis was diagnosed in 58.3% of patients undergoing PUVA therapy (n = 7) and 18.2% of those treated with narrowband UVB 311 nm radiation (n = 6). In turn, data reported by Urgancı Tatlıı and Bilgin [62] showed a positive effect of NB-UVB phototherapy on the severity of *Demodex* sp. infestation. The study group consisted of patients with various skin conditions, such as PsO (n = 9), vitiligo (n = 9), parapsoriasis (n = 6), pruritus (n = 4), mycosis fungoides (n = 3), alopecia totalis (n = 1), hypertrophic lichen planus (n = 1), pityriasis rubra pilaris (n = 1), and pityriasis lichenoides chronica (n = 1).

In the present study, in the group of the 17 AD patients undergoing PUVA/UVB phototherapy, symptomatic *D. folliculorum* infestation was observed in two individuals (11.8%). In turn, symptomatic *Demodex* infestation was observed in three (23.1%) of the 13 AD patients who did not receive PUVA/UVB therapy. Demodicosis was diagnosed in five (25.0%) of the 20 PsO patients treated with phototherapy. All of these patients, testing positive for *Demodex* mites, had ocular symptoms, primarily *blepharitis*, which resolved after demodicosis-targeted treatment. Although the statistical analysis did not reveal a significant relationship between the PUVA/UVB therapy and the prevalence of *Demodex* mites in the AD and PsO patients, the results should not be considered conclusive due to the limited size of the study group. Undoubtedly, physicians prescribing phototherapy and treating patients undergoing the therapy should be aware of the potential risk of the development of secondary demodicosis in this group [61,62].

Direct diagnostic methods for detection of *Demodex* spp. are usually based on the analysis of material collected from the patient, such as eyelashes, facial epidermis scrapings, under a light microscope. In addition to DME, standardized skin surface biopsy (SSSB) is another validated diagnostic methods for *Demodex* spp. [63]. Less frequently, in vivo confocal microscopy (IVCM) or histopathological examination of a skin biopsy are used [64,65]. The sensitivity of these methods in determining *Demodex* mite density is 80.0% (DME) and 37.1% (SSSB) [63]. The sensitivity of IVCM ranges from 51.35% to 83.35% [64]. Importantly, sensitivity is strictly associated with researchers’ experience in using a particular method [64]. In the present research, we excluded the SSSB method because it consists of the use of cyanoacrylate glue, which could have resulted in allergic contact dermatitis in the AD and PsO patients [66] (own observations).

Videodermoscopy [67] or polymerase chain reaction-PCR [68] can also be used to diagnose *Demodex* infestation. However, like in IVCM, these methods are used less frequently and require greater financial outlays. Our previous experience [57] indicates that the most effective method for detecting *Demodex* mites is to collect scrapings from nasolabial folds and hairs from eyebrows or eyelashes. Relying on only one type of sample significantly reduces the likelihood of detecting the parasite. Therefore, in our studies, samples were collected from both the face and eyelashes to minimize the risk of false negative results.

All the patients with *Demodex* mites detected in the skin sample also tested positive for *Demodex* (+) in the eyelash samples. These data suggest that hair follicles located on the eyelid margins may be a common microhabitat for these mites in the analyzed patient group. Pathogenic microorganisms, e.g., those from the genera *Staphylococcus*, *Bacillus*, and *Streptococcus*, are probably involved in the pathogenesis of ophthalmic lesions associated with demodicosis, especially *blepharitis*, which may complicate treatment and contribute to the chronic nature of these lesions [46,69,70,71].

The present study did not show a significant statistical relationship between patients’ sex and the prevalence of *Demodex* sp. in the control group and in the AD and PsO groups, which indicates that sex is not a key determinant of the prevalence of these parasites in AD and PsO patients.

In the studied group of patients with inflammatory skin diseases, the age differences between the *Demodex* (+) and non-infested patients were statistically significant, as the mites were detected more frequently in patients over 49 years of age. The increased risk of *Demodex* infestation with age observed by many researchers may be associated with immunosenescence and changes in the skin composition and function. For example, skin thickness, lipid composition, and sebum production may support *Demodex* colonization [36,72,73].

The present results confirm that *Demodex* sp. infestation is not directly associated with the frequency of exacerbations of inflammatory skin diseases, i.e., AD and PsO, which are characterized by periods of exacerbations and remissions. These findings are consistent with data reported by other researchers, indicating that the *Demodex* colonization of adult skin is a common phenomenon, regardless of skin health status [74,75].

The analysis of the present data revealed no significant differences between the prevalence of *Demodex* sp. in the AD and PsO patients and the disease frequency in the control group. However, as in the general population, these mites may be a proinflammatory factor exerting an impact on the course of the aforementioned chronic diseases [76,77,78]. Serum IgD, α1-antitrypsin, and α1-antichymotrypsin have been detected on the surface of *Demodex* mites, indicating that they are recognized by the immune system [79]. It has also been shown that chitin, a structural component of the *Demodex* skeleton, can be bound by pattern recognition receptors (TLR-2), thereby inducing the secretion of proinflammatory cytokines from keratinocytes [80]. *Demodex* mites exert an indirect effect on skin function, as dead mites release additional immunomodulatory factors into skin appendages, i.e., components of the cell wall of bacteria that are part of their microbiota [81].

Based on genetic testing of skin and nostril swabs from patients, Edslev et al. [82] found that the prevalence of *Demodex* DNA in AD patients was four times higher than in the control group. Topical therapy with glucocorticoids significantly increased the *Demodex* prevalence in the AD patients. The researchers suggested that, given the frequency of this type of treatment in AD patients, *Demodex* mites may have an impact on the clinical picture of the disease. Since the *Demodex* colonization was studied with an indirect method, Edslev et al. [82] did not determine the infestation intensity in the patients with AD.

In this study, the average *Demodex* sp. infestation intensity in the AD and PsO patients and in the control group was similar, which indicates that these inflammatory diseases did not cause an excessive increase in the number of *Demodex* mites present in the pilosebaceous units.

However, it is important to note the possibility of symptomatic infestations in AD and PsO patients even with a relatively low number of mites and the potential comorbidity of demodicosis with primary inflammatory skin diseases.

In the control group, 21.2% of the study participants experienced ophthalmic symptoms, and their prevalence in the AD patients was similar, i.e., 27%. Ophthalmic symptoms are an important component of the clinical picture of AD; nevertheless, it is important to exclude demodicosis in AD patients, who should be tested for the presence of *Demodex* sp., especially when ocular symptoms persist despite treatment and/or recur.

Lesions in patients with AD often develop on the face. A higher rate of ophthalmic symptoms was observed in this group of patients than in the PsO patients and the control group, which is consistent with findings reported by other authors [83,84,85,86,87].

In this study, *Demodex* sp. infestation combined with inflammatory lesions in facial skin was observed in 13.3% of the AD patients, which may probably be ascribed to the constant exposure of this body region to external factors and contact with other people’s skin, e.g., while hugging or kissing. The genetic studies of the diversity of *D. folliculorum* infesting humans have shown the most frequent transmission of these mites between related individuals, i.e., parents and children [88].

In the PsO patients, the rate of *Demodex* sp. infestation combined with ophthalmic symptoms (12.1%) exhibited a similar frequency to that in the AD patients (12.5%). This is probably related to the fact that skin lesions in PsO also involve the periorbital region, which may favor *Demodex* sp. colonization. In turn, the coexistence of *Demodex* sp. with facial skin lesions or ophthalmic symptoms in the control group was observed significantly less frequently, i.e., in 1.5% and 4.5% of the study participants, respectively.

The results of the present study indicate the need for a personalized approach to patients with inflammatory skin diseases, e.g., AD and PsO, in order to eliminate potential proinflammatory factors that can modify the clinical picture in these diseases.

Study strengths: This study directly investigates the relationship between *Demodex* infestation and common inflammatory skin conditions, including atopic dermatitis (AD) and psoriasis (PsO), which are of clear clinical relevance. It also considers additional risk factors for *Demodex* colonization, such as age, sex, use of emollients, and phototherapy. The findings of this study may have direct implications for clinical practice in patients with inflammatory skin diseases. Reporting prevalence rates and mean infestation intensities provides measurable outcomes, while the inclusion of a control group without inflammatory skin disease facilitates relevant comparisons.

Study limitations: Several aspects of the study warrant further exploration in future research. While the present work focuses on emollient use and phototherapy, other therapeutic approaches, such as corticosteroids and biologics, may also influence *Demodex* proliferation and should be systematically evaluated. Increasing the size of specific subgroups (e.g., patients treated with phototherapy) would improve the statistical power of the findings, as some results remain inconclusive due to the limited sample size.

Future studies should also examine the interactions between *Demodex* spp., the skin barrier, the microbiome, and the immune system in patients with AD and PsO. Longitudinal research is particularly important to determine whether *Demodex* infestation precedes disease flares or exacerbates the clinical course of inflammatory skin conditions. Furthermore, data regarding disease severity, measured by the Scoring Atopic Dermatitis (SCORAD) index and the Psoriasis Area and Severity Index (PASI), were not collected in this study. This limitation is acknowledged and should be addressed as a priority in future research.

## 5. Conclusions

Since the prevalence and intensity of *Demodex* infestation in AD and PsO patients did not differ significantly from the control group, it can be assumed that the inflammatory skin diseases do not significantly limit *Demodex* colonization.

*Demodex* infestation is more frequently observed in patients with AD who experience facial lesions. In this group of patients, *Demodex* colonization may contribute to the exacerbation of AD symptoms. *Demodex* mites are detected more frequently in PsO patients with lesions in the periorbital region; therefore, testing for the presence of these mites should be part of the diagnostic process in patients with PsO. *Demodex* spp. infestation should be suspected in AD and PsO patients whose facial skin lesions, including ophthalmic ones, do not respond to treatment targeted specifically at these conditions.

The use of emollients that are part of the treatment of AD and PsO does not significantly increase in the risk of *Demodex* colonization of patients’ skin.

## Figures and Tables

**Table 1:** Characteristics of the study and control groups according to sex, age, and hospitalization.

Variable		AD		PsO		Control	
		n = 30		n = 54		n = 66	
		n	%	n	%	n	%
Sex	Woman	17	56.7	24	44.4	37	56.1
	Man	13	43.3	30	55.6	29	43.9
Age (years)	M ± SD	34.3 ± 14.4		46.6 ± 13.0		44.8 ± 18.6	
	Me (min.–max.)	31.0 (18–80)		45.5 (23–77)		49.0 (20–76)	
Hospitalization	Yes	15	50.0	37	68.5	2	3.0
	No	15	50.0	17	31.5	64	97.0

AD—atopic dermatitis, PsO—psoriasis, n—number of patients in the study group, M—mean, SD—standard deviation, Me—median, min.–max.—minimal and maximal values.

**Table 2:** Prevalence of *Demodex* sp. in patients with different disease onset times in the AD group and the result of the ML chi-square test (n = 29).

*Demodex* sp.	Disease Onset						χ^2^	df	*p*
	In Childhood (0–12 yrs.)		In Adolescence (>12–21 yrs.)		In Adulthood (>21 yrs.)				
	n	%	n	%	n	%			
Yes	2	11.8	1	12.5	1	25.0	0.43	2	0.8078
No	15	88.2	7	87.5	3	75.0			
Total	17	100.0	8	100.0	4	100.0			

χ^2^—chi-square statistic value, df—degrees of freedom, *p*—probability level.

**Table 3:** Prevalence of *Demodex* sp. in patients with different disease onset times in the PsO group and the result of the ML chi-square test (n = 54).

*Demodex* sp.	Disease Onset						χ^2^	df	*p*
	In Childhood (0–12 yrs.)		In Adolescence (>12–21 yrs.)		In Adulthood (>21 yrs.)				
	n	%	n	%	n	%			
Yes	0	0.0	3	25.0	7	20.0	3.23	2	0.1993
No	7	100.0	9	75.0	28	80.0			
Total	7	100.0	12	100.0	35	100.0			

χ^2^—chi-square statistic value, df—degrees of freedom, *p*—probability level.

**Table 4:** Prevalence of *Demodex* sp. in patients with different disease exacerbation rates in the AD group and Fisher’s exact test results (n = 26).

*Demodex* sp.	Frequency of Disease Exacerbation				*p*
	Once a Month or More		Several Times a Year		
	n	%	n	%	
Yes	2	9.5	1	20.0	0.4885
No	19	90.5	4	80.0	
Total	21	100.0	5	100.0	

**Table 5:** Prevalence of *Demodex* sp. in patients with different disease exacerbation rates in the PsO group and the result of the chi-square test with Yates’ correction (n = 50).

*Demodex* sp.	Frequency of Disease Exacerbation				χ^2^	df	*p*
	Once a Month or More		Several Times a Year				
	n	%	n	%			
Yes	2	20.0	7	17.5	0.08	1	0.7825
No	8	80.0	33	82.5			
Total	10	100.0	40	100.0			

χ^2^—chi-square statistic value, df—degrees of freedom, *p*—probability level.

**Table 6:** Logistic regression results for the independent variable: *Demodex* mites in eyelashes and skin of study participants.

n = 150	B	SE	Wald Test	*p*	OR	−95% CI OR	+95% CI OR
Absolute term	−3.00	0.67	20.34	<0.0001	0.050	0.013	0.183
age (years)	0.03	0.01	6.62	0.0101	1.033	1.008	1.059
sex	−0.26	0.21	1.58	0.2089	0.769	0.510	1.159
immunosuppressive medications	−0.40	0.25	2.53	0.1118	0.669	0.408	1.098

B—logistic regression coefficient, SE—standard error, *p*—probability level, OR—odds ratio, CI—confidence interval.

**Table 7:** Prevalence of *Demodex* sp. in patients subjected and non-subjected to PUVA/UVB therapy in the AD group and the result of Fisher’s exact test (n = 30).

*Demodex* sp.	PUVA/UVB Therapy				*p*
	Yes		No		
	n	%	n	%	
Yes	3	23.1	2	11.8	0.6278
No	10	76.9	15	88.2	
Total	13	100.0	17	100.0	

**Table 8:** Prevalence of *Demodex* sp. in patients subjected and non-subjected to PUVA/UVB therapy in the PsO group and the result of the chi-square test with Yates’ correction (n = 54).

*Demodex* sp.	PUVA/UVB Therapy				χ^2^	df	*p*
	Yes		No				
	n	%	n	%			
Yes	5	14.7	5	25.0	0.33	1	0.5635
No	29	85.3	15	75.0			
Total	34	100.0	20	100.0			

χ^2^—chi-square statistic value, df—degrees of freedom, *p*—probability level.

**Table 9:** Prevalence of *Demodex* sp. in patients with and without ophthalmic symptoms in the AD group and Fisher’s exact test results (n = 30).

*Demodex* sp.	Ophthalmic Symptoms				*p*
	Yes		No		
	n	%	n	%	
Yes	1	12.5	4	18.2	0.5500
No	7	87.5	18	81.8	
Total	8	100.0	22	100.0	

**Table 10:** Prevalence of *Demodex* sp. in patients with and without ophthalmic symptoms in the PsO group and results of the chi-square test with Yates’ correction (n = 54).

*Demodex* sp.	Ophthalmic Symptoms				χ^2^	df	*p*
	Yes		No				
	n	%	n	%			
Yes	4	12.1	6	28.6	1.34	1	0.2470
No	29	87.9	15	71.4			
Total	33	100.0	21	100.0			

χ^2^—chi-square statistic value, df—degrees of freedom, *p*—probability level.

## Data Availability

The original contributions presented in this study are included in the article. Further inquiries can be directed to the corresponding authors.
